# Effect of the side of presentation in the visual field on phase-locked and nonphase-locked alpha and gamma responses

**DOI:** 10.1038/s41598-022-15936-7

**Published:** 2022-08-01

**Authors:** Esteban Sarrias-Arrabal, Ruben Martín-Clemente, Alejandro Galvao-Carmona, María Luisa Benítez-Lugo, Manuel Vázquez-Marrufo

**Affiliations:** 1 Lab B508 (Psychophysiology Unit), Experimental Psychology Department, Faculty of Psychology, University of Seville, Seville, Spain; 2 Signal Processing and Communications Department, Higher Technical School of Engineering, University of Seville, Seville, Spain; 3 Department of Psychology, Universidad Loyola Andalucía, Seville, Spain; 4 Physiotherapy Department, Faculty of Nursing, Physiotherapy and Chiropody, University of Seville, Seville, Spain

**Keywords:** Cognitive neuroscience, Neuronal physiology, Visual system, Neurophysiology

## Abstract

Recent studies have suggested that nonphase-locked activity can reveal cognitive mechanisms that cannot be observed in phase-locked activity. In fact, we describe a concomitant decrease in nonphase-locked alpha activity (desynchronization) when stimuli were processed (alpha phase-locked modulation). This desynchronization may represent a reduction in “background activity” in the visual cortex that facilitates stimulus processing. Alternatively, nonphase-locked gamma activity has been hypothesized to be an index of shifts in attentional focus. In this study, our main aim was to confirm these potential roles for nonphase-locked alpha and gamma activities with a lateralized Go/NoGo paradigm. The results showed that nonphase-locked alpha modulation is bilaterally represented in the scalp compared to the contralateral distribution of the phase-locked response. This finding suggests that the decrease in background activity is not limited to neural areas directly involved in the visual processing of stimuli. Additionally, gamma activity showed a higher desynchronization of nonphase-locked activity in the ipsilateral hemisphere, where the phase-locked activity reached the minimum amplitude. This finding suggests that the possible functions of nonphase-locked gamma activity extend beyond shifts in attentional focus and could represent an attentional filter reducing the gamma representation in the visual area irrelevant to the task.

## Introduction

The electroencephalographical signal (EEG) can be analyzed by different technical approaches that allow for studying modulations in time or frequency domains. In the second case, time-frequency methods allow for observing the temporal dynamics across the spectral bands compared to other frequency analyses^1–4^. Regarding time-frequency methods, multiple possibilities exist to observe and analyze the EEG signal. One of these methods is the temporal spectral evolution (TSE)^5,6^. The TSE obtains information about phase-locked and nonphase-locked activity^3^.TSE has been scarcely applied, but it has provided interesting results in healthy and pathological populations^7–10^. The TSE has shown altered or compensatory mechanisms hidden from the phase-locked parameters^7–12^.

Independent of the type of analysis applied, a considerable body of literature has tried to associate the spectral bands analyzed in this study (alpha and gamma) with sensory and/or cognitive processes. With respect to the alpha band, there are few hypotheses about its functional role: an indicator of neural resting^13^, inhibitory control and timing of sensory processing^14^ (synchronization) or a decrease in “neural noise to improve stimulus processing^10^ (desynchronization). Regarding the latter, some studies have described a positive correlation between worse performance in patients with multiple sclerosis and lower desynchronization of alpha activity^10,15,16^. Regarding gamma activity, it has been related to several cognitive processes, including visual binding^17–20^ and attentional processes^21^, such as shifts in attentional focus^10^ or as an attentional filter due to its fine temporal tuning for neural firing (10–30 ms time precision)^20^.

Regarding the Go/NoGo task, previous studies have analyzed phase-locked and nonphase-locked modulations of the alpha band describing similar topographies between both activities (phase-locked and nonphase-locked)^8,9^. However, these studies applied a central stimulation in the Go/NoGo task. Therefore, it was not possible to analyze whether the nonphase-locked activity was modulated by retinotopic features, as has been described for phase-locked activity in previous studies^22–26^. With respect to phase-locked activity, prior studies have reported that P1 and N1 ERPs change topographies as a function of stimuli position (in the right or left visual field)^27,28^. In regard to nonphase-locked activity, two possibilities can arise in the present study: on the one hand, the nonphase-locked alpha activity could be localized in homologous areas of the phase-locked modulation, but on the other hand, the distribution could be wider and extended in both hemispheres, evidencing a general reduction in neural noise in the visual cortex.

Another aim of this study was to evaluate whether the nonphase-locked gamma response is strictly related to the translation of the attentional focus as described in a previous study^10^ or whether it could represent other cognitive mechanisms. To assess this hypothesis, the cognitive task used in the current experiment does not have spatial cues that can engage the readiness of the attentional focus as it occurred in the attention network test. Moreover, the experimental subjects had to move their attentional focus trial by trial for incoming stimuli that were randomly displayed. Our prediction is that nonphase-locked gamma activity would not be synchronized in the same way as was observed in the attention network test.

## Methods

### Participants

Twenty subjects (12 women and 8 men) were selected to participate in the experiment. The ages ranged from 20 to 52 years (mean 35.75, SD 10.73). Eighteen subjects were right-handed. This study was performed in compliance with the Helsinki Declaration. Informed consent was signed by all participants before their inclusion. The Ethics Committee of the University of Seville approved the protocol (project code: PSI2016-78133-P).

### Cognitive task

**Figure 1:**
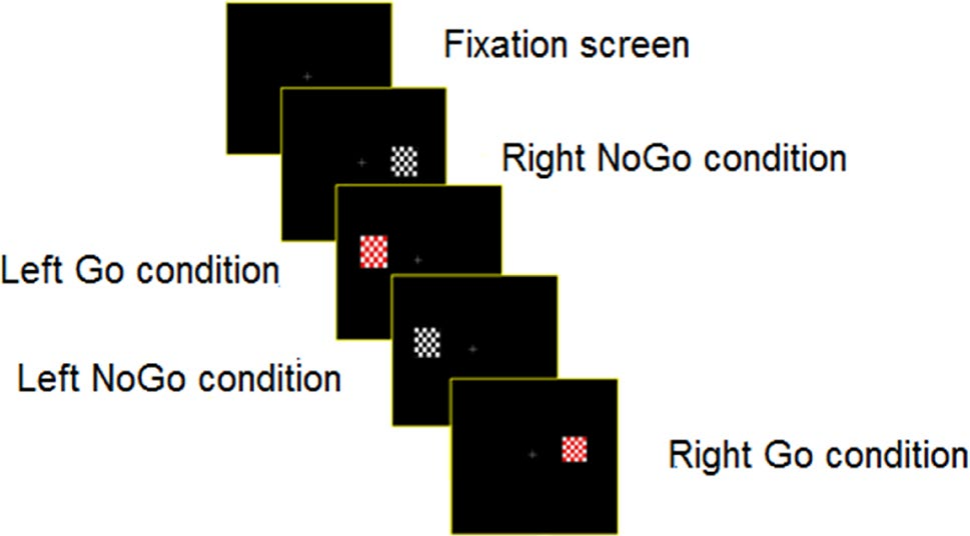
Experimental task (the Go/NoGo paradigm). Four conditions randomly occurred during the experiment. The stimulus duration was 500 ms, and the SOA interval was 1000 ms.

The task was performed inside a faraday chamber. Participants were in front of a computer monitor in which stimuli created by E-prime 2.0 (Psychology Software Tools, Inc., Pittsburgh, PA) were presented. The cognitive task used was a “lateralized Go/NoGo” task in which the subject had to respond to the target (probability: 50%) and ignore stimuli standards (probability: 50%) (Fig. 1). To avoid changes in eye position during the interval intertrial, a fixation cross was present in the middle of the screen. Target and standard stimuli consisted of a rectangle with a checkerboard pattern of the same size. The only difference was in colors. Whereas the target stimulus had red and white squares, the standard stimulus was black and white. The stimulus (target and standard) position was 7.98° × 9.42° visual angle at a viewing distance of 70 cm. Both stimuli were displayed pseudorandomly and presented in the left or right visual field one at a time. Participants had to press left/right mouse buttons with their left/right thumbs when the target stimuli were displayed in the left/right visual field. All stimuli were presented for 500 ms, and the stimulus onset asynchrony (SOA) was 1000 ms, during which the subject could respond. The experiment consisted of 240 trials assembled in one block. All these trials were used to obtain good performance in a target/standard task. The reaction time and percentage accuracy were calculated for both visual fields together. All the participants responded as accurately and quickly as possible.

### EEG recording and processing

To obtain EEG data, a scalp recorder with 58 electrodes (Ag/AgCl) in standard locations of a 10-10 system^29^ and BrainAmp amplifiers (Brain Products GmbH, Germany) were used. Vertical electrooculograms (VEOGs) and horizontal electrooculograms (HEOGs) were also recorded with a bipolar montage. Trials with an HEOG signal outside the ±50 µV range were rejected. During the experiment, the EEG signal was filtered (from 0.01 to 100 Hz), digitized (500 Hz) and stored using Brain Vision Recorder software (Brain Products GmbH, Germany). The reference online was placed in the auricular lobes, and the posterior analysis was run as a common averaged reference. During all the experiments, the impedance was below 5 kOhm. The EEG signal was preprocessed by removing blinking artifacts by ocular correction using the algorithm developed by^30^. After that, the signal was segmented in intervals of 1500 ms (from -500 to 1000 ms with zero being the onset of stimuli) to avoid edge artifacts in the spectral modulations studied^31^. The signal was segmented depending on the position of the target (left or right) to check the effects of the visual field. A baseline correction (-200 to 0 ms) was also applied to both conditions.

After preprocessing, the following steps were run to obtain the phase-locked activity: ERPs were obtained by averaging the signal segmented, and then the averaged was filtered for alpha and gamma bands (8–13 Hz and 30–45 Hz, respectively), rectified to obtain phase-locked activity and finally, a baseline correction (-200 to 0 ms)^8^. For the analysis of the nonphase-locked activity, the temporal spectral evolution (TSE) method was performed before with the following steps: (1) filtering the signal segmented (described above in the preprocessing paragraph) in alpha and gamma bands, (2) rectifying the resulting signal, (3) averaging the EEG epochs, and (4) applying a baseline correction (− 200 to 0 ms). After temporal spectral evolution, subtraction of the phase-locked activity from the TSE signal was subsequently performed to calculate the nonphase-locked response^8^.

**Figure 2:**
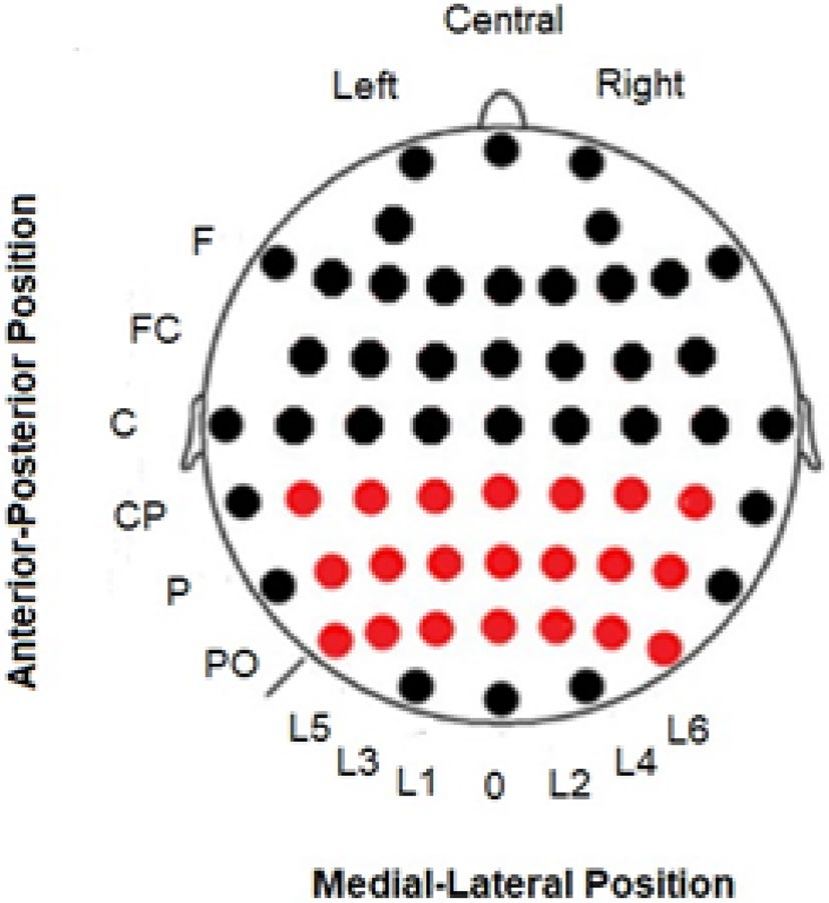
Electrode matrix selected to analyze spectral activity.

Following the guidelines proposed by^32^, the latency for both conditions (left and right visual field presentation) was calculated at the electrode with the maximum amplitude in the grand average of the target conditions. The latency peak was determined individually for each participant in the contralateral derivation of the presentation of the stimuli (PO6 and PO5 for the left and right presentations, respectively). Moreover, the amplitude of the phase-locked and nonphase-locked activity was analyzed at different intervals poststimulus. The intervals were different for the alpha and gamma bands. In the case of alpha, the amplitude was analyzed in the 130–155 ms and 175–260 ms intervals in the phase-locked and nonphase-locked activity, respectively. These intervals included the latency at which both conditions (left/right visual field presentation) reached their maximum amplitude values. In addition, the mean amplitude value in the 360–550 ms interval was analyzed in the nonphase-locked activity because both conditions (left/right) showed a similar decrement in nonphase-locked alpha activity after the first valley. The mean amplitude values were exported for the entire interval in a matrix of 3 × 7 electrodes that covered the posterior area of the scalp in both bands (Fig. 2).

**Figure 3:**
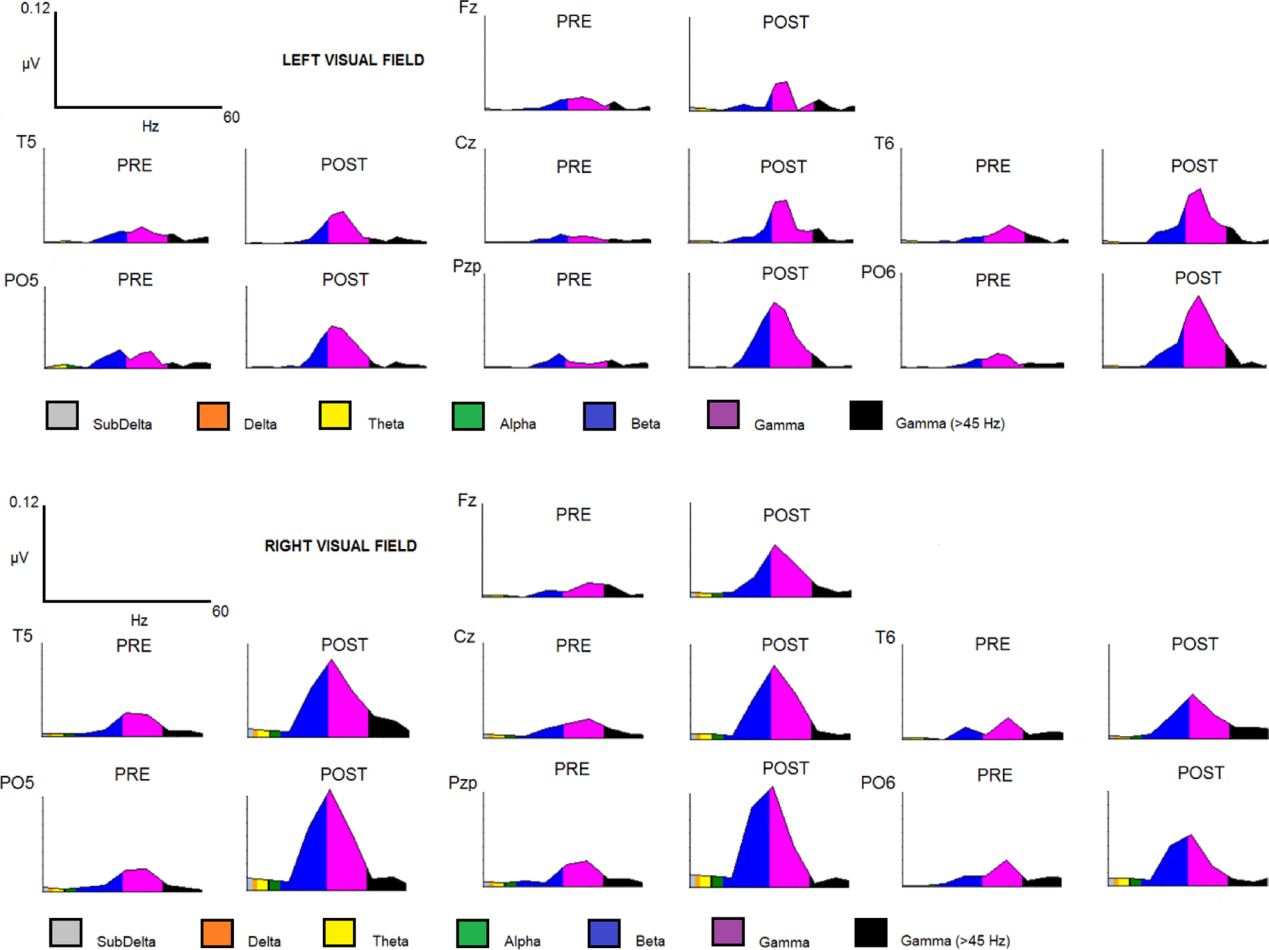
FFT power spectrum for the LVF and RVF conditions.

For the gamma band, the latency peak of gamma activity was determined individually for each participant in the same electrodes depending on the visual field (PO6 for the left visual field and PO5 for the right visual field). The amplitude was analyzed in the 90–130 ms and 60–130 ms intervals for the phase-locked and nonphase-locked gamma activity, respectively. As the alpha band, the mean amplitude values were exported with the same matrix of 3 × 7 used for the alpha band (Fig. 2). We chose those posterior electrodes as ROIs because the maximum amplitude was found in posterior channels for the alpha and gamma bands. In addition, we expected an occipital topography because the task contains visual stimuli. Finally, we calculated the spectral power of gamma (before the TSE) to show that this activity was mainly focused in the posterior electrodes and clearly increased after the stimuli onset (Fig. 3) and discarded the possibility that it might be the refection of the saccade and large distal electromyography (EMG) instead of the neural oscillations.

### Phase analysis for phase-locked and nonphase-locked activity

The nonphase-locked modulation was checked to discard the potential contribution of phase-locked activity over nonphase-locked activity. This analysis was intended to ensure that the nonphase-locked activity was nonphase-locked activity for alpha and gamma. Additionally, we sought to discard any gamma band that was a harmonic from alpha checking if the value phase-locked of the phase-locked activity was different between the alpha and gamma bands. To achieve both goals, the phase-locked and nonphase-locked responses were estimated from each of the individual trials and then averaged for each subject^33,34^. The process to obtain the phase-locked values of both bands was filtering in alpha (8–13 Hz) and gamma (30–45 Hz) (48 dB/octave, Butterworth) and applying the Hilbert transform to calculate the instantaneous phase.

The phases of alpha activity were measured at 142 ms for phase-locked activity (130–155 ms interval). On the other hand, the alpha phases for nonphase-locked activity were measured at 217 ms (175–260 ms interval). Following the same protocol described for alpha, the phases of gamma activity were 110 ms (90–130 ms interval) for phase-locked activity and 95 ms (60–130 ms interval) for nonphase-locked activity. These latencies correspond to the higher amplitudes in the grand average interval of the phase-locked and nonphase-locked responses for both bands.

## Statistical analyses

### Behavioral responses

A descriptive analysis of the mean and standard deviation was performed for both variables, the reaction time and accuracy. The rate of false alarms was < 1%.

### Alpha band

The latencies of phase-locked and nonphase-locked activities were analyzed together. One ANOVA was carried out with the following factors: visual field factor (levels: left and right) and activity factor (levels: phase-locked and nonphase-locked). Regarding the amplitude, the phase-locked activity of the 130–155 ms interval was analyzed by ANOVA with the following factors: visual field factor (levels: left and right); anterior-posterior factor (levels: centroparietal, parietal and parietal-posterior); and lateral-medial factor (Line 5, Line 3, Line 1, medial, Line 2, Line 4 and Line 6) (see Fig. 2 for the details of the positions). The amplitudes of the nonphase-locked activity (first and second valleys) were analyzed in two independent ANOVAs with the same factors used for the phase-locked activity but at different intervals (175–260 ms and 360–550 ms).

### Gamma band

For gamma, we run ANOVAs as described in the alpha section with the same factors considered for latency and amplitude variables. However, the time intervals for the amplitude effects were different compared to the alpha analyses for the phase-locked and nonphase-locked activities. The amplitude was analyzed in the 90–130 ms and 60–130 ms intervals after the onset stimulus for phase-locked and nonphase-locked activities, respectively.

During all statistical processes, the sphericity was calculated with Greenhouse–Geisser. A *p* value lower than 0.05 was considered statistically significant. Post hoc analyses were performed using Bonferroni correction.

## Results

### Behavioral data

The mean and standard deviation values were 371 and 9.07 for reaction time and 99.78 and SD 0.52 for accuracy, respectively.

### Alpha band

**Figure 4:**
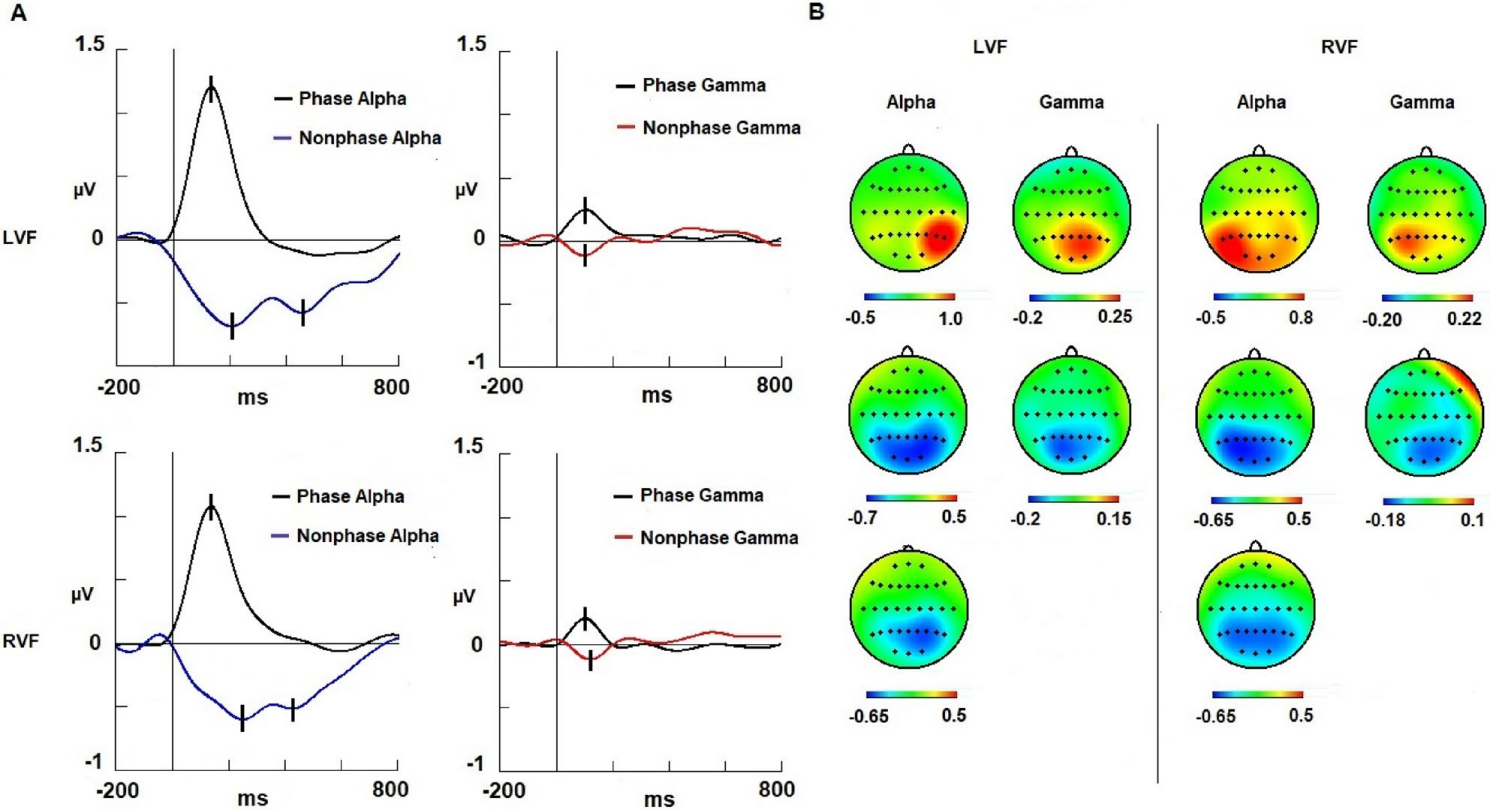
Modulations and topographic maps of the alpha and gamma bands. Spectral activity of the alpha and gamma bands. (**A**) Alpha and gamma modulations are represented in the left column and right column, respectively. (**B**) The upper row represents evoked activity maps for alpha and gamma; the second row shows the first valley of the induced activity maps (alpha and gamma); and the third row displays maps of the second valley of the alpha-induced activity. Nonphase-locked alpha activity was more bilaterally represented than phase-locked activity. The phase-locked gamma response was contralateral and synchronized, whereas nonphase-locked gamma activity was desynchronized in the ipsilateral derivations. *Abbreviations* *LVF* left visual field, *RVF* right visual field, *ms* milliseconds, *µV* microvolts.

With respect to latency, we did not observe differences in the visual field factor (left and right side of the presentation) (F (1,19) = 0.6306; *p* = 0.436) (Fig. 4). In contrast, the activity factor was statistically significant [F (1,19) = 31.513; *p* < 0.001; ŋ^2^: 0.624], with the phase-locked activity being faster (138 ms) than the nonphase-locked activity (218 ms) (Fig. 4). No statistically significant result was found for the interaction of both factors. The latency values are presented in Table 1.

**Table 1:** Latency values for phase-locked and nonphase-locked alpha and gamma bands.

	Latency (mean ± standard deviation)		
	LVF	RVF	P value
**Alpha band**			
Phase-locked	137 ± 24	140 ± 47	1.000
Nonphase-locked	209 ± 75	227 ± 78	1.000
Nonphase-locked	457 ± 79	455 ± 70	1.000
**Gamma**			
Phase-locked	110 ± 51	102 ± 39	1,000
Nonphase-locked	107 ± 46	132 ± 49	1.000

*LVT* left visual field, *RVF* right visual field.

**Figure 5:**
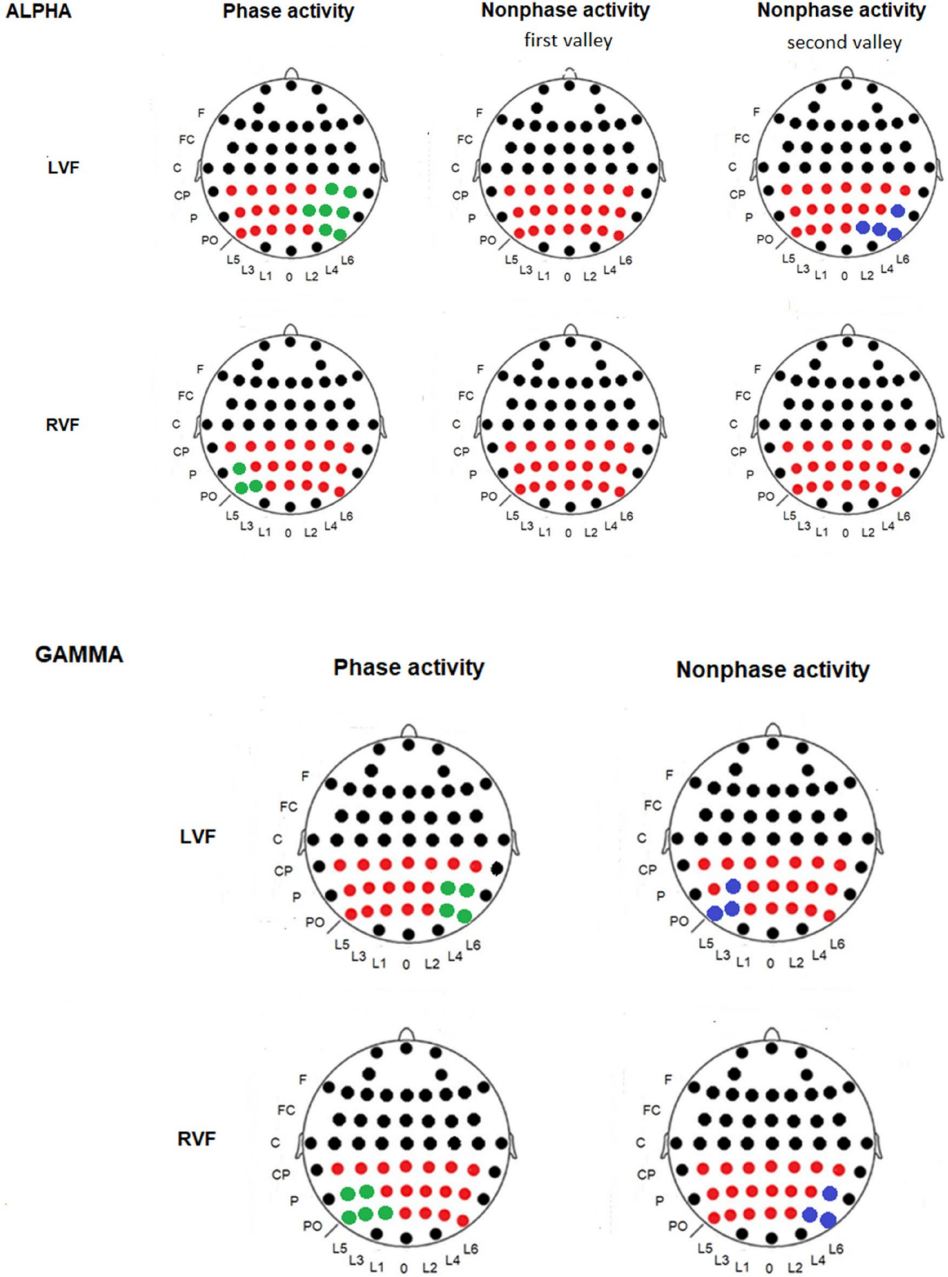
Significantly different electrodes from the alpha and gamma bands. Electrodes showing larger synchronization in the phase-locked response (green) and electrodes representing larger desynchronization in the nonphase-locked response (blue) compared to their homologous electrodes on the contralateral hemisphere. The first and second valleys correspond to different latencies in alpha desynchronization (see Table 1). *Abbreviations* *LVF* left visual field, *RVF* right visual field.

Regarding amplitude in the phase-locked activity, there were no differences for the visual field factor (F (1,19) = 0.59272; *p* = 0.450) in the 130–155 ms interval. However, the interaction “visual field” × “anterior-posterior” × “lateral-medial” showed a statistically significant result [F (12, 228) = 2.786; *p* < 0.001; ŋ^2^: 0.128]. The Bonferroni post hoc test indicated that when the target was displayed on the left side, the amplitude was larger in the electrodes of the right hemisphere than in the electrodes of the left hemisphere (*p* < 0.001) (Fig. 5). Nonetheless, if the target was displayed on the right visual field, the amplitude was larger in the electrodes of the left hemisphere than in the electrodes of the right hemisphere (*p* <0.001) (Fig. 5). In summary, the distribution of the phase-locked activity was contralateral to the position of the target in the visual field.

In the nonphase-locked activity, the ANOVA did not show amplitude differences for the visual field factor (F (1,19) = 0.26435; *p* = 0.613) (or in other interactions where this factor was involved) in the 175–260 ms interval, confirming a bilateral pattern independent of where the target was displayed (left or right visual field). In the 360–550 ms interval after the onset of stimulus, the ANOVA did not show amplitude differences for the visual field actor either (F (1,19) = 0.47277; *p* = 0.500). However, post hoc Bonferroni comparison for the interaction “visual field” × “anterior-posterior” × “lateral-medial” showed that if the target was displayed in the left visual field, the right hemisphere achieved a greater decrease in nonphase-locked activity compared with the left hemisphere (Fig. 5). When the target was displayed in the right visual field, the statistical analysis did not show differences between hemispheres (Fig. 5). The latency and amplitude values are shown in Table 1.

### Gamma band

Similar to the alpha band, we did not observe differences in the latency caused by the visual field factor (left and right) (F (1,19) = 0.8166; *p* = 0.377) (Fig. 2). Moreover, the activity factor for the gamma band did not show significant differences (F (1,19) = 1.5650; *p* = 0.226) (Fig. 3). The latency and amplitude values are shown in Table 1.

Regarding the amplitude, there were no differences for the visual field factor in the 90–130 ms interval for the phase-locked activity (F (1,19) = 1.65380; *p* = 0.213) or in the 60–130 ms interval for the nonphase-locked response (F (1,19) = 0.06116; *p* = 0.807) (Fig. 4). However, the interaction “visual field” × “anterior-posterior” × “lateral-medial” showed statistically significant results in both the 90–130 ms interval (phase-locked activity) [F (12, 228) = 8.455; *p* < 0.001; ŋ^2^: 0.308] and 60–130 ms interval (nonphase-locked activity) [F (12, 228) = 2.515; *p* = 0.003; ŋ^2^: 0.117] (Fig. 2).

With respect to the 90–130 ms interval, the post hoc Bonferroni test showed that if the target was displayed in the left visual field, the phase-locked gamma band reached a larger amplitude in some derivations of the right hemisphere compared to the left hemisphere (Fig. 4).On the other hand, when the target was displayed in the right visual field, the statistical analysis found a larger amplitude of phase-locked activity in some leads of the left hemisphere compared to the right hemisphere (Fig. 3).

Strikingly, in the nonphase-locked gamma activity, we observed a different pattern compared to the nonphase-locked alpha activity. In the 60–130 ms interval, the Bonferroni post hoc test showed that if the target was displayed in the left visual field, the left hemisphere reached a higher desynchronization in some electrodes of activity in contrast to the right hemisphere (Fig. 4). On the other hand, when the target was displayed in the right visual field, the statistical analysis found higher desynchronization of activity in some derivations of the right hemisphere in comparison to the left hemisphere (Fig. 5).

### Phase analysis for nonphase-locked and phase-locked activity

**Figure 6:**
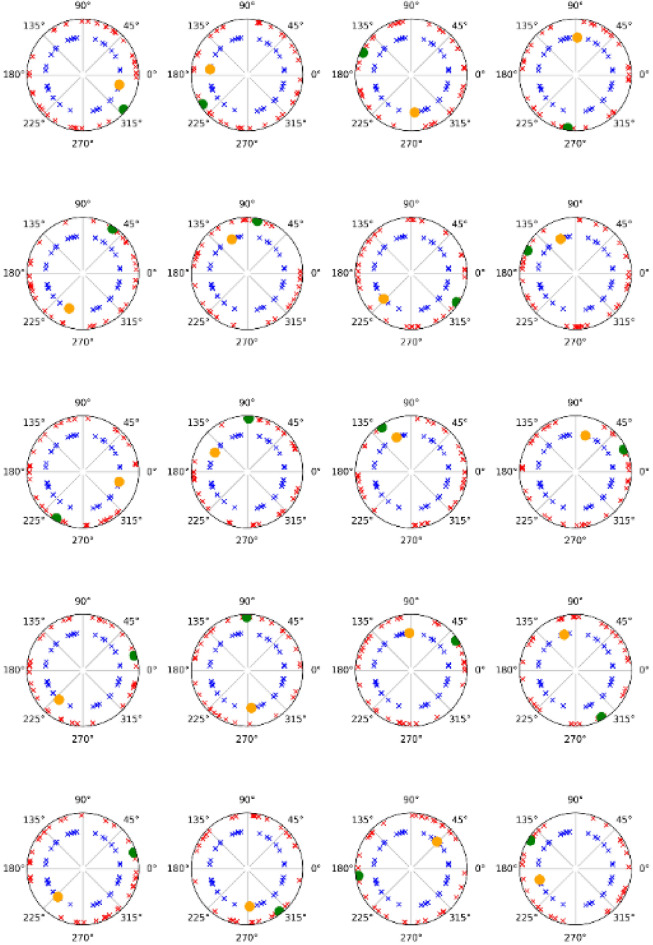
Phase values of the alpha band. The phase-locked values of the alpha band for phase-locked and nonphase-locked activities following the presentation of the target (20 subjects). The green dots and red crosses represent phase-locked and nonphase-locked activity (respectively) when the target is displayed in the left visual field. Yellow dots and blue crosses are when the target was displayed in the right visual field. The individual representations demonstrate that nonphase-locked values are randomly distributed in all subjects and are not centered around the values of phase-locked activity.

**Figure 7:**
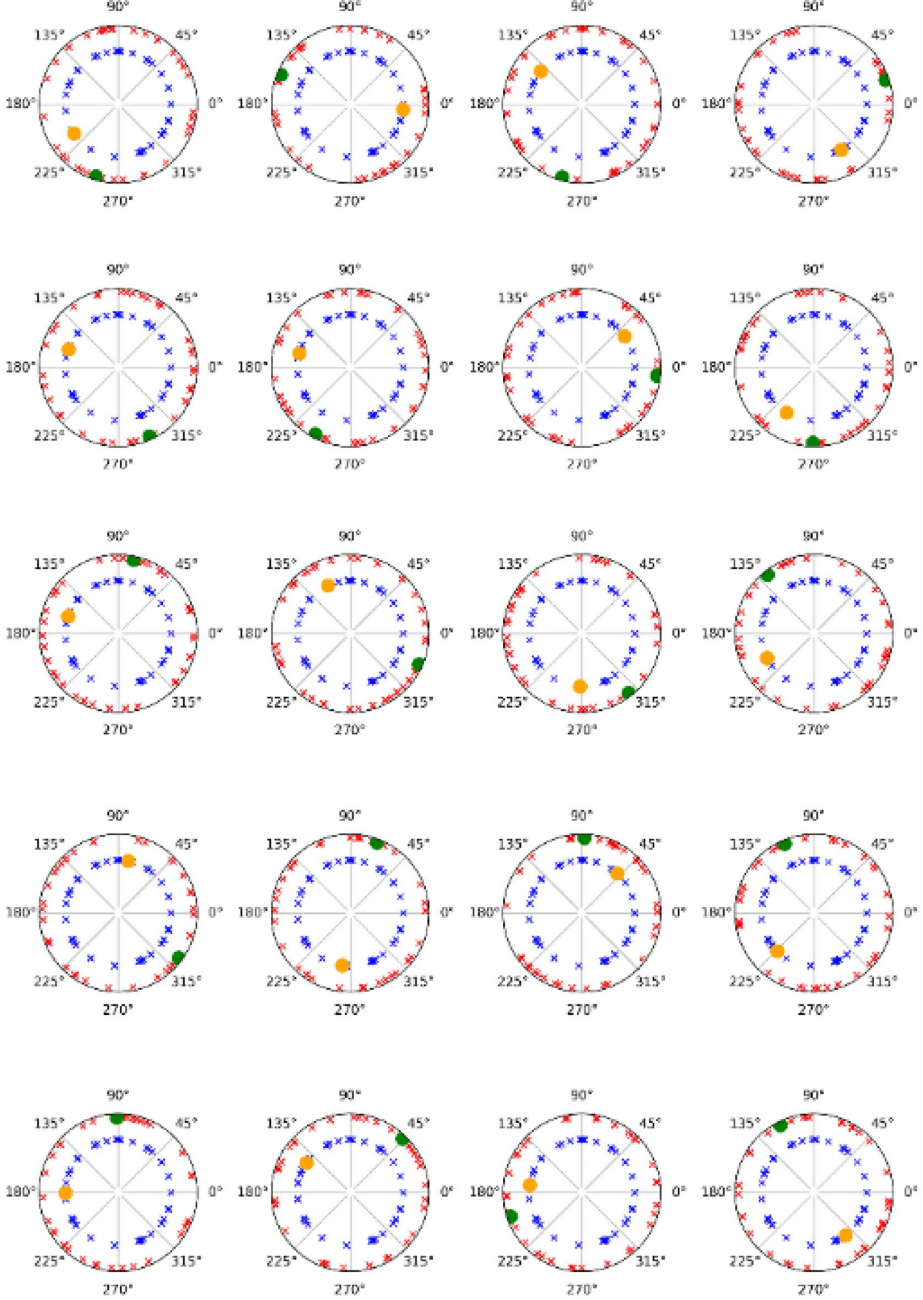
Phase values of the gamma band. The phase-locked values of the gamma band for phase-locked and nonphase-locked activities following the presentation of the target (20 subjects). Green dots and red crosses represent phase-locked and nonphase-locked activity (respectively) when the target is displayed in the left visual field. Yellow dots and blue crosses are when the target is displayed in the right visual field. As indicated in Fig. 4, plotting the individual representations demonstrates the dissociation between both activities (phase-locked and nonphase-locked).

The results of phase-locked analyses for phase-locked and nonphase-locked let us discard the potential contribution of the phase-locked activity over the nonphase-locked activity, as has been described in previous studies^8,10,33^ (Figs. 6, 7).

## Discussion

### Behavioral responses

The behavioral data analysis showed that the mean reaction time (371 ms) was similar to previous studies^8,35,36^ that used a Go/NoGo paradigm with healthy controls. The subjects showed accuracy near 100% (99.78%), and false alarms were below 1%. These parameters indicated that performance was appropriate in this experimental group with a good balance of response speed and accuracy.

### Alpha activity

Regarding the alpha modulations, there was a delay in the latency in which the nonphase-locked activity reached the highest desynchronization compared to the latency in which the phase-locked activity reached the highest synchronization in the alpha band (Fig. 4). The relative timing between both activities suggests that both modulations are independent, as no difference was found in a previous study^8^. It seems that phase-locked responses are more consistent, whereas nonphase-locked responses have a wider range of latency values.

However, latency is not the only parameter of these modulations that supports the dissociation of both activities. Regarding the scalp distribution, phase-locked alpha (synchronization) topography showed a contralateral pattern of activity with respect to the presentation of the target on the visual field (Fig. 5). Some authors have proposed that phase-locked alpha activity at latencies within the 100–200 ms interval may be a spectral correlate of early ERPs (P1 and N1)^14,37,38^. On the other hand, it is well known that the P1/N1 components show a contralateral distribution in this type of visual task^27,28^. Therefore, those studies and our results support the hypothesis that the early phase-locked alpha activity may represent the spectral content of early ERPs (P1 and N1) and have a predominantly contralateral distribution.

With respect to nonphase-locked alpha activity, the scalp distribution of the first valley showed a bilateral distribution (Fig. 4).In previous studies, we interpreted this to mean that it is necessary to decrease the “neural noise” of alpha in the visual cortex to allow sensory or cognitive processes operating in alpha or other bands to perform their function^8,10^. The similarity of the maps found for phase-locked and nonphase-locked alpha modulations was interpreted as a specific reduction of the nonphase-locked alpha activity in similar areas where a phase-locked response was present. Therefore, the bilateral topography of nonphase-locked alpha signals in the current study suggests that the decrease in neural noise would not occur exclusively in homologous areas of the visual cortex. Note that in previous studies, we used a central Go/NoGo so that it was not uncommon to observe a bilateral pattern of activity for both phase-locked and nonphase-locked activities^8,10^.

The second valley showed a topographic pattern different from that of the first valley (Fig. 4). A contralateral distribution was observed when the target was displayed on the left visual field, whereas the topographical distribution was bilateral when the target was presented on the right visual field (Figs. 4 and 5). This result has been interpreted as an asymmetric monitorization of visual stimuli with dominance of the right hemisphere when the attentional system is involved^22–25^. Finally, it is difficult to associate cognitive function with such a long latency (> 300 ms) in this paradigm. However, it is possible to conclude that this activity is related to other cognitive mechanisms compared to the first nonphase-locked response attending to the different latency and topography. Therefore, we discarded the hypothesis that the second valley necessarily represents a rebound of the activity of the first valley, as has been proposed previously^10^.

### Gamma activity

With respect to the gamma band, there were no significant differences between the peak latency of the phase-locked activity and the valley latency of the nonphase-locked modulation (Fig. 4). This result does not contradict the suggestion proposed in the alpha section about the independency of both domains (phase-locked and nonphase-locked). It is likely that phase-locked and nonphase-locked activities can play their roles in close latencies depending on the spectral bands and/or demands of the task.

Regarding the topography, the phase-locked gamma band exhibited contralateral activity to visual field presentation, the same as in the alpha band (Figs. 4, 5). We suggest that the gamma band may represent part of the spectral content of P1 and N1 ERPs at early latencies (< 200 ms), mainly P1^37–40^. However, some authors reject this suggestion because gamma has the lowest amplitude and may be insufficient to generate ERP components^41^. Nonetheless, it is possible that the gamma response represents other aspects of visual processing added to the alpha modulation in short latencies. Future studies are necessary to precisely define their functional roles in phase-locked responses.

In the nonphase-locked activity, the gamma band did not show a bilateral pattern of activity like the first valley of alpha (Fig. 4). Instead, the desynchronization of nonphase-locked activity was ipsilateral to the location of the target on the visual field (Figs. 4, 5). This pattern contrasts with the contralateral activity with respect to the attended side of the phase-locked gamma band (Figs. 4, 5). These findings about phase-locked and nonphase-locked gamma activity would fit well with the idea that gamma may act as an attentional filter^20^. In the current results, our interpretation is that the higher the desynchronization of nonphase-locked gamma, the greater the filtering of the visual processing occurs in that hemisphere. An attentional filter in the early steps of visual processing has been described in previous studies for phase-locked responses^41–44^, and the current study extends this proposal to nonphase-locked modulations in the EEG.

Note that in this study, the nonphase-locked gamma activity showed desynchronization instead of synchronization, which was shown in previous studies with the attention network test (ANT)^10^. Applying ANT, we proposed that nonphase-locked gamma activity represented shifts in the attentional focus. However, the gamma band may represent other processes in a lateralized Go/NoGo for several reasons. First, the latency in which nonphase-locked gamma reached the maximum synchronization in ANT was longer^10^ than the latency in which nonphase-locked gamma reached the maximum desynchronization in this task (lateralized Go/NoGo). Second, nonphase-locked gamma activity showed a larger amplitude in the ANT^10^ than nonphase-locked gamma activity in lateralized Go/NoGo. Third, nonphase-locked gamma activity had a bilateral topography in the ANT^10^, whereas the nonphase-locked gamma activity was ipsilateral to the attended side in this study. Fourth, nonphase-locked gamma activity showed synchronization in the ANT^10^ in contrast to the nonphase-locked gamma activity in Go/NoGo, which showed desynchronization. It seems reasonable to state that nonphase-locked modulations are dependent on the task and have specific cognitive roles that are different (but somehow linked) to the phase-locked responses.

It is relevant to highlight that the present results are focused on low gamma activity (from 30 to 45 Hz). Other potential bands have been defined in gamma activity involved in other cognitive processes^45–48^. The present results suggest the participation of the low gamma in attentional filtering outside of other potential roles as an index of the translation of attentional focus in visual space^10^. The present and previous studies confirmed that gamma modulations could play diverse roles in cognitive processing, and more specific studies are required to define the complex panorama of this spectral activity.

Finally, the results from the phase-locked analysis discard any contribution of phase-locked activity from nonphase-locked activity (phases randomly represented) (Figs. 6, 7). In addition, both bands showed different values in both activities (phase-locked and nonphase-locked) at the latencies of interest. Therefore, we can affirm that both activities (phase-locked and nonphase-locked) in both bands (alpha and gamma) may represent independent cognitive processes.

## Conclusion

Nonphase-locked alpha activity could represent a decrease in neural noise that is not circumscribed to the areas for phase-locked responses. Moreover, the second valley of nonphase-locked alpha activity seems to represent different cognitive mechanisms than the first valley, attending to the latency and topography, which rejects the hypothesis of a simple rebound of this alpha activity. Last, nonphase-locked gamma modulation could represent an index of attentional filtering in the first steps of visual processing, and it is not only involved in the translation of the attentional focus.

## Data Availability

The datasets generated and/or analyzed during the current study are available from the corresponding author upon reasonable request.

## References

[1] Boashash B (2016). Time-Frequency Signal Analysis and Processing: A Comprehensive Reference.

[2] Pfurtscheller G, Neuper C, Mohl W (1994). Event-related desynchronization (ERD) during visual processing. Int. J. Psychophysiol..

[3] Vázquez Marrufo M, Vaquero E, Cardoso MJ, Gómez C (2001). Temporal evolution of α and β bands during visual spatial attention. Cogn. Brain Res..

[4] Vázquez Marrufo M, Vaquero Casares E, Cardoso Moreno MJ, Gómez González CM (2001). Dinámica temporal de la frecuencia del electroencefalograma. Metodología y aplicaciones. Rev Neurol..

[5] Lehtelä L, Salmelin R, Hari R (1997). Evidence for reactive magnetic 10-Hz rhythm in the human auditory cortex. Neurosci. Lett..

[6] Salmelin R, Hari R (1994). Spatiotemporal characteristics of sensorimotor neuromagnetic rhythms related to thumb movement. Neuroscience..

[7] Sarrias-Arrabal E, Eichau S, Galvao-Carmona A, Domínguez E, Izquierdo G, Vázquez-Marrufo M (2021). Deficits in early sensory and cognitive processing are related tophase and nonphase EEG activity in multiple sclerosis patients. Brain Sci..

[8] Vázquez-Marrufo M, García-Valdecasas M, Caballero-Diaz R, Martin-Clemente R, Galvao-Carmona A (2019). Multiple evoked and induced alpha modulations in a visual attention task: Latency, amplitude and topographical profiles. PLoS ONE..

[9] Vázquez-Marrufo M, Caballero-Díaz R, Martín-Clemente R, Galvao-Carmona A, González-Rosa JJ (2020). Individual test-retest reliability of evoked and induced alpha activity in human EEG data. PLoS ONE.

[10] Vázquez-Marrufo M, Sarrias-Arrabal E, Martin-Clemente R, Galvao-Carmona A, Navarro G, Izquierdo G (2020). Altered phase and nonphase EEG activity expose impaired maintenance of a spatial-object attentional focus in multiple sclerosis patients. Sci. Rep..

[11] Ciesielski KT, Hämäläinen MS, Geller DA, Wilhelm S, Goldsmith TE, Ahlfors SP (2007). Dissociation between MEG alpha modulation and performance accuracy on visual working memory task in obsessive compulsive disorder. Hum. Brain Mapp..

[12] Lasaponara S, Pinto M, Aiello M, Tomaiuolo F, Doricchi F (2019). The hemispheric distribution of α-Band EEG activity during orienting of attention in patients with reduced awareness of the left side of space (Spatial Neglect). J. Neurosci..

[13] Pfurtscheller G, Maresch H, Schuy S (1977). Inter- and intrahemispheric differences in the peak frequency of rhythmic activity within the alpha band. Electroencephalogr. Clin. Neurophysiol..

[14] Klimesch W, Sauseng P, Hanslmayr S (2007). EEG alpha oscillations: The inhibition–timing hypothesis. Brain Res. Rev..

[15] Keune PM (2017). Exploring resting-state EEG brain oscillatory activity in relation to cognitive functioning in multiple sclerosis. Clin. Neurophysiol..

[16] Kiiski H (2012). Only low frequency event-related EEG activity is compromised in Multiple Sclerosis: Insights from an independent component clustering analysis. PLoS ONE..

[17] Mably AJ, Colgin LL (2018). Gamma oscillations in cognitive disorders. Curr. OpinNeurobiol..

[18] Tallon-Baudry C, Bertrand O, Delpuech C, Pernier J (1996). Stimulus specificity of phase-locked and nonphase-locked 40 Hz visual responses in human. J. Neurosci..

[19] Gruber T, Giabbiconi CM, Trujillo-Barreto NJ, Müller MM (2006). Repetition suppression of induced gamma band responses is eliminated by task switching. Eur. J. Neurosci..

[20] Tallon-Baudry C (2009). The roles of gamma-band oscillatory synchrony in human visual cognition. Front. Biosci..

[21] Gruber T (1999). Selective visual-spatial attention alters induced gamma band responses in the human EEG. Clin. Neurophysiol..

[22] Berchicci M (2019). Electrophysiological evidence of sustained spatial attention effects over anterior cortex: Possible contribution of the anterior insula. Psychophysiology..

[23] Di Russo F (2012). Spatiotemporal brain mapping of spatial attention effects on pattern-reversal ERPs. Hum. Brain Mapp..

[24] Di Russo F (2021). Sustained visuospatial attention enhances lateralized anticipatory ERP activity in sensory areas. Brain Struct. Funct..

[25] Di Russo F (2021). Modulation of anticipatory visuospatial attention in sustained and transient tasks. Cortex..

[26] Vázquez-Marrufo M, del Barco-Gavala A, Galvao-Carmona A, Martín-Clemente R (2021). Reliability analysis of individual visual P1 and N1 maps indicates the heterogeneous topographies involved in early visual processing among human subjects. Behav. Brain Res..

[27] Clark VP, Fan S, Hillyard SA (1994). Identification of early visual evoked potential generators by retinotopic and topographic analyses. Hum. Brain Mapp..

[28] Clark VP, Fannon S, Lai S, Benson R (2001). Paradigm-dependent modulation of event-related fMRI activity evoked by the oddball task. Hum. Brain Mapp..

[29] American Electroencephalography Society (1994). Guideline thirteen. J. Clin Neurophysiol..

[30] Gratton G, Coles MG, Donchin E (1983). A new method for off-line removal of ocular artifact. Electroencephalogr. Clin. Neurophysiol..

[31] Cohen, M. X. *Analyzing Neural Time Series Data: Theory and Practice* Illustrated edn (MIT Press, 2014).

[32] Keil A, Müller MM (2010). Feature selection in the human brain: Electrophysiological correlates of sensory enhancement and feature integration. Brain Res..

[33] David O, Kilner JM, Friston KJ (2006). Mechanisms of evoked and induced responses in MEG/EEG. NeuroImage..

[34] Truccolo WA, Ding M, Knuth KH, Nakamura R, Bressler SL (2002). Trial-to-trial variability of cortical evoked responses: Implications for the analysis of functional connectivity. Clin. Neurophysiol..

[35] Cassidy SM, Robertson IH, O’Connell RG (2012). Retest reliability of event-related potentials: Evidence from a variety of paradigms. Psychophysiology..

[36] Vázquez-Marrufo M, González-Rosa JJ, Galvao-Carmona A, Hidalgo-Muñoz A, Borges M, Peña JLR, Izquierdo G (2013). Retest reliability of individual p3 topography assessed by high density electroencephalography. PLoS ONE.

[37] Gruber WR (2005). Alpha Phase Synchronization predicts P1 and N1 latency and amplitude size. Cereb. Cortex.

[38] Hanslmayr S (2007). Alpha phase reset contributes to the generation of ERPs. Cereb. Cortex..

[39] Sauseng P (2007). Are event-related potential components generated by phase resetting of brain oscillations? A critical discussion. Neuroscience.

[40] Herrmann CS, Lenz D, Junge S, Busch NA, Maess B (2004). Memory-matches evoke human gamma-responses. BMC Neurosci..

[41] Luck SJ (1995). Multiple mechanisms of visual-spatial attention: Recent evidence from human electrophysiology. Behav. Brain Res..

[42] Luck SJ, Ford MA (1998). On the role of selective attention in visual perception. Proc. Natl. Acad. Sci. USA.

[43] Luck SJ, Woodman GF, Vogel EK (2000). Event-related potential studies of attention. Trends Cogn. Sci..

[44] Luck SJ, Yard SAH (1995). The role of attention in feature detection and conjunction discrimination: An electrophysiological analysis. Int. J. Neurosci..

[45] Murty DVPS, Shirhatti V, Ravishankar P, Ray S (2018). Large visual stimuli induce two distinct gamma oscillations in primate visual cortex. J. Neurosci..

[46] Han C, Shapley R, Xing D (2021). Gamma rhythms in the visual cortex: functions and mechanisms. Cogn. Neurodyn..

[47] Han C, Wang T, Yang Y, Wu Y, Li Y, Dai W (2021). Multiple gamma rhythms carry distinct spatial frequency information in primary visual cortex. PLoS Biol..

[48] Wang B, Han C, Wang T, Dai W, Li Y, Yang Y (2021). Superimposed gratings induce diverse response patterns of gamma oscillations in primary visual cortex. Sci. Rep..

